# A Computational Theory of Mindfulness Based Cognitive Therapy from the “Bayesian Brain” Perspective

**DOI:** 10.3389/fpsyt.2020.00404

**Published:** 2020-05-15

**Authors:** Zina-Mary Manjaly, Sandra Iglesias

**Affiliations:** ^1^Department of Neurology, Schulthess Clinic, Zurich, Switzerland; ^2^Department of Health Sciences and Technology, ETH Zurich, Zurich, Switzerland; ^3^Translational Neuromodeling Unit (TNU), Institute for Biomedical Engineering, University of Zurich and ETH Zurich, Zurich, Switzerland

**Keywords:** Mindfulness Based Cognitive Therapy (MBCT), being mode, decentering, cognitive reactivity, Bayesian brain, predictive coding, active inference

## Abstract

Mindfulness Based Cognitive Therapy (MBCT) was developed to combine methods from cognitive behavioral therapy and meditative techniques, with the specific goal of preventing relapse in recurrent depression. While supported by empirical evidence from multiple clinical trials, the cognitive mechanisms behind the effectiveness of MBCT are not well understood in computational (information processing) or biological terms.

This article introduces a testable theory about the computational mechanisms behind MBCT that is grounded in “Bayesian brain” concepts of perception from cognitive neuroscience, such as predictive coding. These concepts regard the brain as embodying a model of its environment (including the external world and the body) which predicts future sensory inputs and is updated by prediction errors, depending on how precise these error signals are.

This article offers a concrete proposal how core concepts of MBCT—(i) the being mode (accepting whatever sensations arise, without judging or changing them), (ii) decentering (experiencing thoughts and percepts simply as events in the mind that arise and pass), and (iii) cognitive reactivity (changes in mood reactivate negative beliefs)—could be understood in terms of perceptual and metacognitive processes that draw on specific computational mechanisms of the “Bayesian brain.” Importantly, the proposed theory can be tested experimentally, using a combination of behavioral paradigms, computational modelling, and neuroimaging. The novel theoretical perspective on MBCT described in this paper may offer opportunities for finessing the conceptual and practical aspects of MBCT.

## Introduction

Mindfulness Based Cognitive Therapy (MBCT) is an evidence-based psychotherapeutic approach that was designed as a treatment for relapse prevention after repeated episodes of depression (1, 2). MBCT was originally developed based on theoretical concepts about the origin of depression and potential mechanisms of relapse (3–5). One central idea in these concepts concerns cognitive reactivity as a risk factor for relapse of depression. In brief, in periods of low mood, negative thinking patterns that are associated with negative emotions and painful bodily sensations are thought to be reactivated automatically and may lead to relapse of depression by self-reinforcing cycles of ruminative thinking; this, in turn, is assumed to strengthen the association between dysphoria and depressogenic thinking and to increase the probability that ruminations are triggered in future episodes of low mood (1, 6, 7). In order to interrupt and prevent these processes, a core strategy of MBCT is the cultivation of mindfulness skills, derived from Buddhist traditions (8–11), which are supposed to target this self-perpetuating process.

The clinical efficacy of MBCT for preventing relapse after three or more depressive episodes has been demonstrated by multiple randomized control trials (6, 7, 12–19). By contrast, so far, the mechanisms and factors that mediate the effects of MBCT are not fully understood, nor whether these mechanisms match those of the theory behind MBCT [for review, see (20)]. As for other psychotherapeutic approaches (21), a precise understanding of cognitive and neurophysiological mechanisms that mediate therapeutic effects are important in order to predict and optimize treatment outcomes, guide treatment selection for individual patients, and finesse existing therapeutic approaches (21).

This article offers a novel perspective on potential mechanisms of MBCT that derive from computationally inspired theories of brain function. Specifically, the view presented in this paper draws upon Bayesian concepts of perception and action that feature prominently in contemporary cognitive neuroscience (22–25). In the following, we refer to these concepts as the “Bayesian brain hypothesis” (26). These theories assume that the brain constructs a model of “the world” (i.e., the physical and social environment, but also the body) which guides both perception and action. In particular, this article refers to two concepts: predictive coding which regards perception as Bayesian inference under a hierarchical model about the world (27, 28); and active inference that explains action selection as a belief-fulfilling process (29–31). These approaches are united by one overarching idea: that the brain’s overall goal is to minimize surprise (or prediction error) about sensory inputs (22, 32).

There are several reasons why it seems useful to examine MBCT from the perspective of the Bayesian brain hypothesis. First, Bayesian brain theories formalize and make testable predictions about cognitive processes that are of relevance for understanding the human mind in health and disease. Generally, the investigation of mental disorders by Bayesian models of cognition has become a very active field of research [for reviews, {25, 31, 33, 34)].

Second, key central constructs of the cognitive theories behind the development of MBCT—including the “being mode,” decentering, and reactivity (2, 4)—can be understood in terms of processes inherent to Bayesian theories of cognition. This article explicates these conceptual bridges in order to provide a complementary perspective on mechanisms of MBCT.

Third, this novel perspective facilitates linking MBCT to neurophysiological mechanisms. This is because numerous neurophysiological studies have begun testing key mechanisms proposed by the Bayesian brain hypothesis. For example, studies using functional magnetic resonance imaging (fMRI) and electroencephalography (EEG) have demonstrated that brain activity contains signals which reflect processes as mathematically predicted by Bayesian brain theories (35–38). Given a link between Bayesian concepts of cognition and MBCT, neurophysiological readouts of this sort might become useful as markers of treatment outcome in MBCT and may complement classical self-report measures (e.g., questionnaires) and clinical interviews in this regard.

This article is not the first to address the question whether potential mechanisms of contemplative practices, including mindfulness, could be related to “Bayesian brain” concepts; in particular, see Farb et al. (39) and Lutz et al. (40). Farb et al. (39) examined mindfulness as one example of how contemplative practices could be understood in terms of predictive coding. Lutz et al. (40) discussed how an influential phenomenological model of focused attention meditation (41) could be recast in terms of active inference.

By contrast, this article focuses on MBCT as a specific psychotherapeutic approach to depression. To the best of our knowledge, this paper (and the thesis it is based on; (42) represents a first attempt to understand therapeutic mechanisms of MBCT in terms of concrete processes predicted by the Bayesian brain hypothesis. In order to keep the article accessible for a general readership, this article restricts itself to a conceptual analysis and keeps mathematic formulations to a minimum. The reader who is interested in detailed mathematical treatments of the processes discussed is referred to existing literature (27, 43, 44).

The article is structured as follows. Following a brief review of the theoretical foundations of MBCT, it summarizes the general idea behind the Bayesian brain hypothesis and explains some of the key terms and concepts. We subsequently introduce a Bayesian perspective on MBCT that relates some of the key concepts in MBCT (doing and being modes, decentering, reactivity) to processes proposed by Bayesian theories of cognition. The final section discusses how the implications of the Bayesian perspective on MBCT could be tested in experimental studies and how this may help improve clinical practice.

## Mindfulness Based Cognitive Therapy

Depression is one of the world’s leading causes of disability (45, 46). It frequently displays a chronic course, with multiple occurrences of recovery (remission) and recurrence (relapse) (47). MBCT was developed as a therapy to prevent relapse in patients with previous episodes of depression (1, 2). This development was based on cognitive models of vulnerability to depression, in particular, the theory of Interacting Cognitive Subsystems (ICS) (48–50) and the Differential Activation Hypothesis (3, 51, 52). In addition, ideas and concepts from Buddhist philosophy played an important role for the development of mindfulness-based interventions, e.g., Mindfulness-Based Stress Reduction (MBSR) (10) and subsequently MBCT (1, 6). For detailed accounts, the interested reader is referred to (1, 4, 6).

### Pathomechanistic Concepts of Depression Relapse in MBCT

During depressive episodes, low mood frequently coexists with negative thinking patterns, painful emotions, and disturbing body sensations (53). Following successful remission, the probability of future relapse is high but difficult to predict for individual patients (54–57). Importantly, the more frequently depressive episodes have been experienced in the past, the more vulnerable the individual becomes to relapse (58).

What causes this vulnerability? Following the work by Teasdale and colleagues (51, 52), MBCT builds on the notion that cognitive reactivity, “the tendency to react to small changes in mood with large changes in negative thinking” (1, p. 30), represents one primary source of vulnerability [for a review of empirical findings, see (59)]. Generally, the theory behind MBCT assumes that the higher the reactivity of an individual, the greater the vulnerability of this individual for recurrences of depressive episodes; this has been confirmed empirically (59). More specifically, in periods of even mild dysphoria, cognitive reactivity is thought to trigger negative thinking patterns that are associated with painful emotions and bodily sensations. These negative thoughts and affective experiences may reinforce each other, resulting in a vicious cycle that ultimately leads to recurrence of depression (5–7). Importantly, with each episode of depression, the associations between depressed mood and negative thinking patterns are thought to be strengthened, increasing the likelihood that depressogenic ruminations are reactivated in future moments of dysphoria. As a consequence, MBCT is based on the premise that the risk of relapse can be reduced if one becomes aware of the reactivation of negative thinking patterns during dysphoria and learns to disengage from the self-reinforcing cycles of ruminations and emotions.

A key concept in MBCT is that cognitive reactivity and the vicious cycle described above is sustained by a particular mode of mind—the so-called “doing mode” [for a summary of concepts of modes of mind in MBCT, see (60)]. This particular mode of mind sets in when negative thinking patterns and associated emotions and bodily sensations are recognised (2, 61).

Generally, the doing mode is activated by a discrepancy between desired and actual experiences (e.g., thoughts, emotions, bodily sensations). Once activated, the doing mode elicits actions that are predicted to minimize this discrepancy, monitors the consequences, and reinstates further action if the discrepancy has not been reduced yet. The doing mode is not pathological per se but represents a goal-oriented mode that draws upon experience and models for predicting the future. It is usually helpful, particularly in application to concrete problems of the external world. “It is natural, then, that we should turn to this same doing mode when things are not as we would like them to be in our *personal, internal* worlds—our feelings and thoughts, or the kind of person we see ourselves to be. And this is where things can go terribly wrong.” (1, p. 68).

In other words, the doing mode can become harmful when applied to problems which we cannot influence or for which there is no immediate solution, or when a perceived discrepancy is assigned emotional significance even though it is just a fleeting event. In these cases, mental activities elicited by the doing mode are futile and get stuck in ongoing monitoring whether perceived discrepancies have decreased; this manifests as ruminations, increases distress, and “binds” the negative emotional state to the thinking pattern (62). When the doing mode becomes problematic in this manner, it is also referred to as the “driven-doing mode” (1, p. 69).

These notions of vulnerability to relapse, based on the concepts of cognitive reactivity and the doing mode, have been guiding the development of MBCT. MBCT brings together techniques for mindfulness meditation—in particular from MBSR (10, 63)—with elements of cognitive therapy (CT). A central aim of MBCT is to increase the patient’s awareness of the rapid, automatic reactivation of negative thinking patterns during moments of dysphoric mood and the unhelpful activation of the doing mode. To achieve this, mindfulness meditation serves to cultivate a different mode of mind, the “being mode.” This mode of mind differs from the doing mode as it does not aim to reach a certain goal but is explorative and experiential (1). In other words, the being mode allows sensations to be as they are—in this moment, without any interpretation or evaluation, and without any urge to change them. It is thus anchored in the present moment whereas the doing mode is required to predict the future and draws from experience in the past. Additionally, the being mode involves a “decentered” perspective on the working of the mind. Decentering, a further core construct of MBCT, means that thoughts, emotions, and sensations are simply observed as they arise and pass, without engaging with them. This enables the individual to view these occurrences simply as temporary and automatic mental events, but not as defining the “self” or constituting any “truth” or “facts” about reality. Decentering is therefore also often referred to as meta-awareness (4, 64, 65).

### Structure of the MBCT Program

MBCT is classically taught as an 8-week program that serves to cultivate one’s ability to activate the being mode and to stimulate the growth of decentering skills. The following represents an extremely brief summary; for a detailed description, see (1).

In MBCT, a variety of formal meditation practices are introduced where the individual pays attention to a particular focus in a non-striving and non-judgmental way. Whenever attention drifts off, the individual is invited to simply acknowledge this mind-wandering and to gently reorient attention back to the previous focus (4). The practices encourage the individual to recognize when the doing mode is taking over and to engage in the being mode instead. Through practice, this approach increasingly allows one to identify the problematic deployment of the doing mode (i.e., driven-doing mode) in everyday life and to disengage from this purposefully in later stages of the program. In all practices, emphasis is placed on the attitude by which attention is redirected, namely in a most gentle, kind, non-judgmental and compassionate fashion. This attitude is particularly important as it allows the individual to turn towards any painful experiences and resist the urge to wish for change and problem solving.

In addition to mindfulness meditation, MBCT incorporates elements of CT, including psychoeducation (for example, that dysphoric mood can trigger negative thinking patterns which, in turn, can trigger emotions and bodily sensation, or vice versa). Additionally, it is emphasized that thoughts are not facts, but rather passing mental events that do not reflect the “self” and which one does not have to identify with. However, there is one fundamental difference between a “classical” CT approach and those CT elements used in MBCT. Whereas regular CT aims to change the *content* of negative thinking, MBCT does not try to alter thought content but its *relationship* with emotion and bodily sensations (1, 4). This derives from the rationale that changing the content effectively requires the use of the goal-oriented doing mode. This is exactly the opposite of what MBCT tries to cultivate—that is, to foster the being mode in general and find a more appropriate balance between doing and being mode.

In summary, central goals of MBCT practice include the reduction of cognitive reactivity—which is thought to convey vulnerability to depressive relapse—and to take a more decentered perspective on transitory mental events such as thoughts, emotions, and bodily sensations. To achieve this, MBCT aims at cultivating the being mode or, more specifically, the flexibility to switch between being mode and doing mode, depending on which has greater adaptive capacity in a given context. Below, we will examine how these core features of MBCT can be understood in terms of processes that play a central role in Bayesian theories of brain function, and how this interpretation may foster a better understanding of the therapeutic effects of MBCT in cognitive and neuronal terms.

### Empirical Evidence

The effectiveness of MBCT for reducing risk of relapse for patients with multiple previous episodes of depression has been demonstrated by several randomized clinical trials (6, 7, 12–15, 18, 19, 66) and meta-analyses (67, 68). By contrast, it is less clear whether the mechanisms of change match those suggested by the theoretical framework behind MBCT—and how this would best be tested using methods beyond subjective self-report. A recent systematic review of variables that might predict or mediate the effects of MBCT on treatment outcome (20) emphasized the need for more rigorous studies to examine causal specificity. Notably, while there are numerous neuroimaging studies on mindfulness [e.g. (69–73)] and comprehensive reviews [e.g. (74, 75)], so far very few empirical studies have specifically examined the neural processes that underpin MBCT (76).

The next section turns to Bayesian theories of cognition and brain function that offer a novel perspective on potential mechanisms of MBCT and could facilitate the design of empirical studies that employ behavioral and neuroimaging readouts.

## The “Bayesian Brain Theory”

The “Bayesian brain theory” has grown in popularity over the last few decades and is presently one of the most influential theories in cognitive neuroscience (for review, see Friston, 22). It refers to the idea that the brain uses probability theory, and specifically Bayes’ theorem, to infer the states of the world that give rise to the sensory inputs it receives.

The use of Bayesian theory for explaining perception has a long history in cognitive science and neuroscience. It dates back to work by Helmholtz in the late 19^th^ century (77) who suggested that perception corresponds to “unconscious inference.” More recently, Gregory (78) used the example of visual illusions to argue that perception could be understood as Bayesian inference. However, this proposal was mathematically informal, and it is only in the past two decades that the Bayesian brain theory evolved into a detailed description of perception. Several recent reviews are available (27, 79).

**Figure 1:**
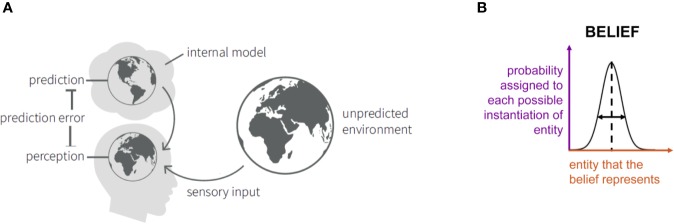
**(A)** Schematic summary of the “Bayesian brain” notion that the brain contains an internal model consisting of beliefs about the states of the environment. These give rise to predictions about sensory inputs. The discrepancy between the actual and the predicted sensory inputs (prediction error) serves to update the model. Adapted from **Figure 3** in Haker et al. (82), with permission. **(B)** An illustration of the concept of “beliefs” as probability distributions. Here, we consider Gaussian probability distributions (or, more precisely, densities) that are characterized by an expectation (or mean; represented by the vertical dashed line) and precision (inverse variance; symbolized by the horizontal double arrow). The x-axis (red) indicates the entity that the belief represents (e.g. the temperature of a particular object). The y-axis (violet) represents, simply speaking, the probability that is assigned to each possible instantiation of this entity (in the above example: the probability that object temperature has a particular value).

According to a Bayesian view on perception, the brain creates and continuously updates a model of the external world (including the physical environment but also the body), based upon past experience and homeostatic needs (25) (**Figure 1A**). This model is necessary for the brain in order to infer the state of the world. This is because the brain has no direct access to the world: the only information it receives are sensory inputs that are both noisy and ambiguous. For example, the visual inputs that the retina receives are ambiguous: the same image can result from a variety of objects when conditions of lightning, visual angle, etc. are changed, and the brain must therefore infer the state of the world that most likely caused the retinal image (80). This inference is thought to rest on Bayes’ theorem and requires knowledge or beliefs about which state is most likely *a priori*. In science, this is also known as an inverse problem: given some observations (sensory inputs), the challenge is to infer what the external causes are (81).

Bayes’ theorem dates back to the 18^th^ century and was developed by the Presbyterian priest Thomas Bayes (83). In a cognitive science context, it can be understood as describing how “beliefs” should be updated when one receives new information. Here, the term “belief” denotes a mental representation that an individual holds and which may reflect prior experience. Beliefs can concern concrete (e.g., physical properties of objects in the world) or abstract (e.g., the intentions of other people) entities of the world. To accommodate inevitable uncertainty, beliefs have a probabilistic representation and correspond to probability distributions; they are thus characterized by statistics like expectation (mean) or precision (inverse variance), see **Figure 1B**. Furthermore, beliefs can depend on each other and collectively constitute a model of the world. For reviews of probabilistic concepts of cognition, see Kersten and Yuille (80), Griffiths et al. (84) and Petzschner et al. (79).

**Figure 2:**
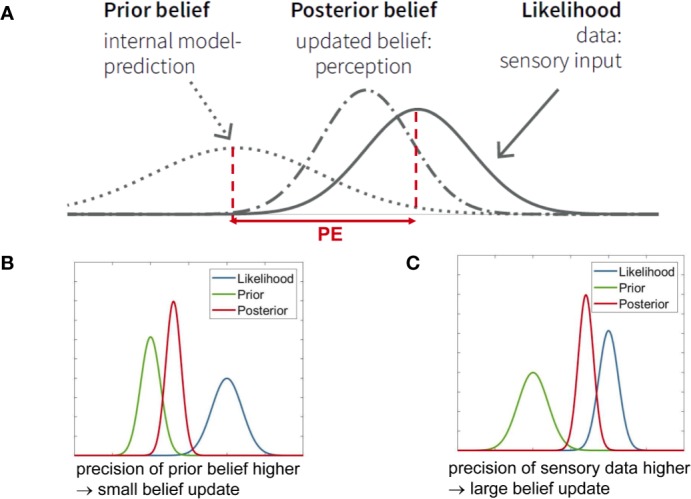
**(A)** Graphical summary of Bayes’ theorem (see Eq. 1) for the case of Gaussian probability distributions. It illustrates that the posterior represents a compromise between prior and likelihood, depending on their relative precision. PE is the abbreviation for “prediction error.” To revisit the example from Figure 1B, let us consider perception of temperature. The actually perceived temperature (posterior belief) is a compromise between the expected or predicted temperature (prior) and the sensory input (likelihood). The posterior belief can also be understood as updating the prior belief, where the magnitude of the belief update depends on the prediction error (PE) and the relative precisions (inverse variance) of the prior and the likelihood. In this example, the precision of sensory input (likelihood) is higher, therefore the posterior is closer to the likelihood. This panel is adapted from **Figure 2** in Haker et al. (82), with permission. **(B)** When the precision of the prior belief is higher than the precision of the data (likelihood), a small belief update results, i.e., the posterior stays close to the prior. **(C)** When the precision of the data (likelihood) is higher than the precision of the prior belief, a large belief update results, i.e., the posterior moves more strongly towards the data.

Concretely, Bayes’ theorem describes how an initial belief (or prior information) about a particular quantity is integrated with or updated by new observations (i.e. sensory input), resulting in an updated belief (or posterior probability); see **Figure 2**. Equivalently, it can be understood as inference about a quantity x, given an initial belief and new observations y. Mathematically, a short form of writing Bayes’ theorem is:

(1) p(x|y)∝p(y|x)p(x)

Here, p(x) represents the “prior”, i.e., information that is available about the quantity x, prior to receiving new information. p(y|x) is the so-called “likelihood” and denotes the new information or data. In the brain, this is equivalent to sensory inputs y caused by the quantity x, as discussed below. Finally, p(x|y) represents the conclusion or inference and is called the “posterior.” Equation 1 says that the posterior probability is proportional to the product of likelihood and prior. For continuous variables, the integral of the right side of Equation 1 (product of likelihood and prior) over x has to equal one; for discrete variables, the same holds for the sum over x. Visually, this means that the larger the width of a distribution, the shorter its height (**Figure 2**). The width of the distribution represents the variance, and the inverse of variance is called precision; this definition holds both for continuous and discrete quantities. As an example, **Figure 1B** shows the expectation (mean) and precision of a continuous quantity x that is normally distributed. Precision can be thought of as the confidence one assigns to a prior belief, or as the information (signal-to-noise ratio) one ascribes to sensory inputs.

Precision plays a central role in Bayesian inference. Bayes’ theorem in Eq. 1 can mathematically also be re-expressed as a precision-weighted belief update. As shown by **Figure 2**, the posterior is always a compromise between the prior and the likelihood, where the relative precision of the two determines the posterior’s shape and location. For example, if the precision of the new data (likelihood) is higher (i.e. narrower curve) than the precision of the prior information, then the posterior moves closer to the likelihood (**Figure 2C**). By contrast, if the precision of the new data is lower than the precision of the prior, the posterior moves closer to the prior (**Figure 2B**). Mathematically, the belief update in Bayes’ theorem depends on the so-called prediction error - the discrepancy between new data and prior belief (i.e. predicted sensory information)—that is weighted by the ratio between data precision and prior belief precision (**Figure 2A**).

A Bayesian view on perception proposes that the incoming sensory information (inputs or sensations) corresponds to the likelihood which is constantly compared to the predicted sensory information. This prediction derives from the prior beliefs encoded in the brain’s model. The difference between the actual and the expected sensory input is the prediction error. The posterior is the actual percept and derives from updating the prior belief by means of a precision-weighted prediction error (**Figure 2**). Furthermore, in the Bayesian brain theory, predictions and prediction errors correspond to quantities that are exchanged between neuronal populations whose activity encodes probability distributions that have a certain precision (27, 43).

An important implication of the Bayesian brain theory is that perception as inference or belief updating corresponds to minimizing the surprise about the sensory inputs (32). Here, “surprise” is a mathematical concept from information theory that is approximated by prediction errors. Intuitively speaking, the better the brain’s model of the world, the more successfully it can predict sensory inputs and the less surprise it experiences.

**Figure 3:**
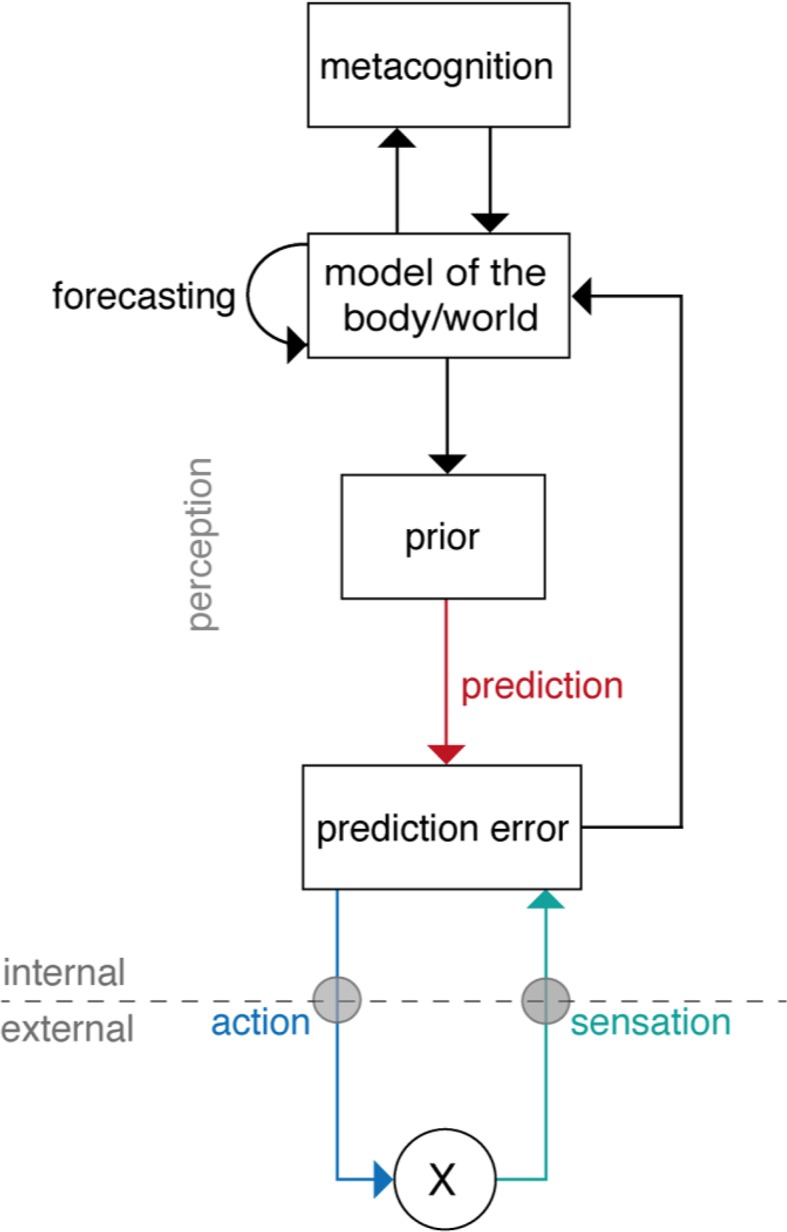
A general scheme of “Bayesian brain” theories of cognition. Here, the overall goal is to minimize prediction errors. Prediction errors represent, simply speaking, the difference between actual sensory input (or sensation, green arrow) and a prediction about the input which originates from a prior belief (red arrow). Minimization of the prediction error can either be achieved by updating the brain’s model (perceptual inference, e.g., according to predictive coding; middle part of figure) or by choosing actions such that beliefs are fulfilled, and the predicted sensory inputs occur (active inference; lower part of figure). In addition to inference and action, hierarchical Bayesian models also allow for forecasting future states and offer an opportunity to integrate metacognition as a top-level that monitors levels of prediction errors (upper part of figure). Reprinted from Petzschner et al. (25), with permission from Elsevier.

Under this notion, the brain has two general strategies to reduce prediction errors and thus surprise (compare **Figure 3**). A first strategy to minimize prediction error is known as *active inference* (22). This refers to acting upon the world (e.g., the body) in order to change it in such a way that sensory inputs become consistent with prior beliefs. This idea is similar to homeostatic regulation, a process that elicits actions whenever sensory inputs deviate from a given setpoint. Indeed, Bayesian formulations in the spirit of active inference exist that describe homeostatic regulation as the reflex-like fulfilment of homeostatic beliefs: these regard setpoints as homeostatic beliefs (about the states the body should inhabit) and describe how those beliefs are “defended,” rather than altered, by eliciting actions whenever prediction errors occur (25, 85). This is particularly important for those homeostatic beliefs which cannot be updated arbitrarily (e.g., body temperature must not exceed a particular limited range). Here, the force of action depends on two things (85). First, on the magnitude of the prediction error: If bodily sensations deviate from the prior belief significantly, the prediction error is large and a significant regulatory action follows (e.g., strong activation of endocrine or autonomic nervous system processes via hypothalamus or brainstem). Second, on the precision of the homeostatic belief: actions scale with the precision of the belief, because a prediction error becomes more meaningful and the need for correction more urgent when homeostatic beliefs are precise (i.e., the permissible homeostatic range is tight).

The second general strategy to minimize prediction errors is *perceptual inference*. Here, as described above, the incoming sensation (likelihood) is compared with the expected sensation (prior belief), and any discrepancy between expected and actual sensation creates a prediction error which is used to update the belief (compare **Figure 2**). This belief update results in an improved model that is less surprised by the sensory input.

A particularly popular implementation of perceptual inference, called “predictive coding” (27, 28), suggests that the brain is hierarchically organized, with increasing levels of abstraction the further one moves up the hierarchy. This idea of a hierarchy in the brain is motivated by neuroanatomical studies that provide evidence for hierarchical relations among cortical areas (86). Predictive coding assumes that at each level of the hierarchy, neuronal populations encode a probability distribution (a prior belief or prediction about the activity of the level below) which is signaled to the level below via top-down connections (28). There, the prediction can be compared to the actual activity level, and a prediction error can be computed that is passed upwards again where it is used to update the belief at the level above. In other words, at each level of the hierarchy, Bayesian inference is performed where the prior is provided by a predictive signal from the level above and the likelihood is represented by the local activity. The greater the magnitude of a prediction error at the bottom of the hierarchy, the further up the hierarchy its effects will percolate and lead to adjustments of the model.

**Figure 4:**
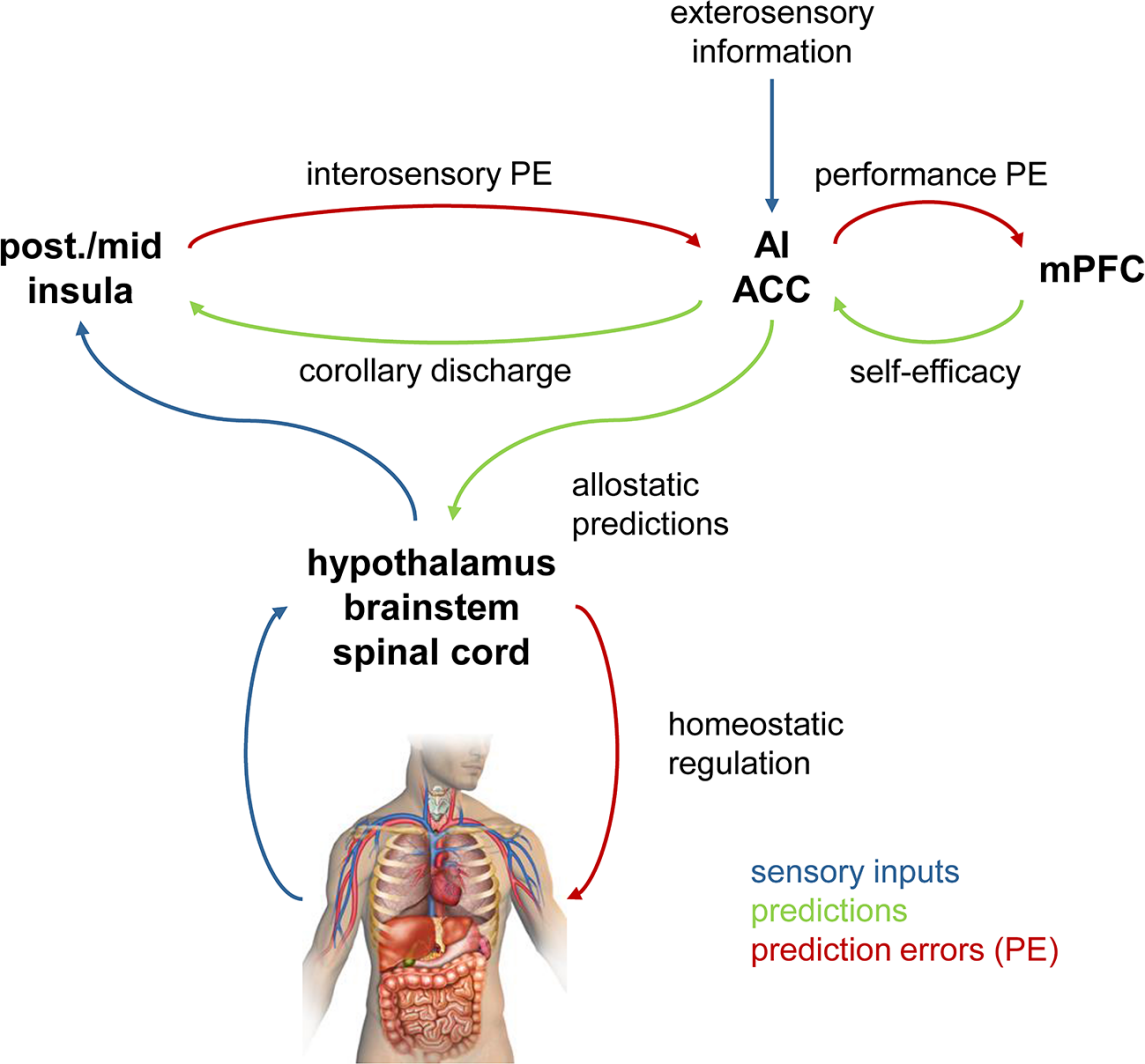
A hypothetical anatomical circuit for homeostatic and allostatic regulation (87). The lower part represents a reflex arc in which homeostatic beliefs about bodily states (represented in hypothalamus, brainstem, spinal cord) are defended (protected) against deviations, by eliciting actions that depend on precision-weighted prediction errors. The upper part represents a cortical hierarchy for perceptual inference that is capable of modulating the homeostatic beliefs via descending connections and can implement anticipatory (allostatic) control. A top metacognitive layer (tentatively assigned to medial prefrontal cortex) holds beliefs about performance levels (i.e., levels of prediction errors at the top of the hierarchy). Colors have the same meaning throughout this figure, as indicated by the legend. It is important to keep in mind that in Bayesian treatments of inference-control loops, the direction in which predictions and prediction errors are signaled reverses when switching from the afferent branch (perception) to the efferent branch (action). For example, in the afferent branch, prediction errors are signaled upwards in the hierarchy, whereas in the efferent branch, they are used by descending projections to inform actions. post., posterior; ACC, anterior cingulate cortex; mPFC, medial prefrontal cortex. Reproduced from Figure 3 in Manjaly et al. (87), with permission from BMJ Publishing Group Ltd.

Importantly, a hierarchical model of this sort can not only implement perceptual inference, but can also accommodate other components of adaptive behavior (see **Figure 3** for a schematic overview and **Figure 4** for a putative anatomical circuit with focus on bodily perception and control). First, a hierarchical architecture can not only infer current states of the world, but also forecast future states, for example, by inferring on *trajectories* of states of the world (88). Second, a hierarchical architecture can implement anticipatory control, also known as allostatic regulation (89), through active inference. As described above, in active inference, the prediction error is not reduced by updating beliefs but by fulfilling beliefs through actions. In a hierarchical model, the beliefs that drive actions are positioned at the bottom of the hierarchy and do not undergo Bayesian belief updating, however, they can be modulated by perceptual inference or forecasts from higher layers (see **Figures 4** and **8**). For example, homeostatic beliefs about bodily states can be shifted or adjusted in their precision by predictions from a perceptual hierarchy (85). This enables anticipatory control or allostatic regulation (89): for example, if forecasts of bodily or environmental processes indicate future violations of homeostasis, homeostatic beliefs can be altered such that actions are elicited in advance which mitigate or avoid the predicted threat (25, 85). This is important for understanding stress-related diseases because subjectively perceived or anticipated threats can lead to activation of sympathetic processes (“fight-flight” responses) over prolonged periods (90, 91). Finally, a hierarchical model can also naturally integrate metacognition, or specifically, self-monitoring of one’s agency in exerting control. In principle, in order to assess how well the brain’s model is capable of inferring states of the world and elicit adequate actions, it is sufficient to monitor a single quantity (prediction error) at the top of the perceptual hierarchy (85). If this top-level prediction error is chronically enhanced, this indicates that the brain’s model is inadequate and provides poor inferences/predictions, and/or that the brain is not capable of eliciting adaptive actions. In either case, chronically enhanced prediction error or surprise signals a loss of control and has been proposed as an index of “learned helplessness” (85).

Importantly, perceptual inference and active inference are applied to both the physical and social environment and to the body (25). In other words, the brain needs to construct a comprehensive model that considers both exteroceptive inputs from the external world and interoceptive inputs from the body. This model is used to achieve the overarching goal of the brain: to minimize prediction errors about both environmental and bodily states, in order to reach a state where incoming sensory inputs lead to minimum surprise.

## Neurophysiological Implications of the Bayesian Brain

**Figure 5:**
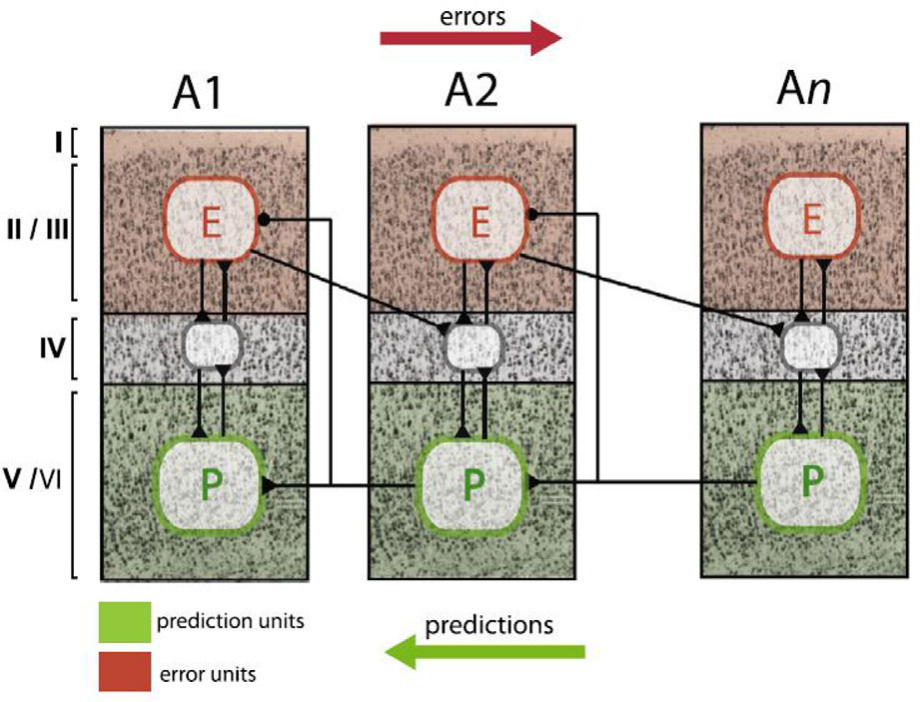
Schematic summary of proposed neurophysiological implementations of hierarchical Bayesian inference in the cortex, specifically, predictive coding. In this scheme, neurons that compute prediction errors (red plates, E) are situated in supragranular layers and signal these errors to neurons in granular layers (grey plates) at the next higher level. By contrast, neurons that compute predictions (green plates, P) are lcoated in infragranular layers and signal these predictions to neurons in both infra- and supragranular layers at the next lower level. This figure is reproduced, with permission, from Heilbron and Chait (95).

The Bayesian brain perspective proposes concrete computational processes how the brain’s model is dynamically adapted from moment to moment. Additionally, it has a plausible biological basis. Friston (27) summarized the available neurobiological evidence and proposed a minimal neuronal model which assigns different functions to different cortical layers; updates can be found in more recent reviews [e.g., (32, 92–94)]. As shown by anatomical tract tracing studies (86), layers II–III have ascending (forward or bottom-up) connections to the granular layers of hierarchically higher areas. Conversely, the infragranular layers have descending (backward or top-down) connections to extragranular layers in hierarchically lower areas. The predictive coding model by Friston (27) proposes that prediction errors are computed by pyramidal cells in supragranular layers II and III. Predictions, on the other hand, are encoded by pyramidal cells in infragranular layers V and VI. Each level of the hierarchy consists of these neuronal units with their intrinsic connections between layers and extrinsic connections between areas. This architecture allows for signaling of predictions and prediction errors in order to implement Bayesian inference (compare **Figures 2** and **5**).

However, as was outlined above, belief updates in Bayesian inference are dependent on the magnitude of precision. The higher the precision of the prediction (or prior belief), the smaller the belief update; conversely, the higher the precision of the sensory input, the larger the belief update (see **Figure 2**). Neurophysiologically, precision is thought to be encoded by neuromodulatory transmitters, for example dopamine and acetylcholine, which shape the excitability of neurons and thus impact on the variance of their activity (32).

In summary, the Bayesian brain theory represents an integrative model of how perception and action are implemented by the brain in order to fulfil a homeostatic principle: the minimization of prediction error or surprise. In this manner, it provides a bridge from theoretical models of cognition to physiological models of brain processes and suggests concrete neurobiological and computational mechanisms. The next section will examine how the ideas underlying the Bayesian brain theory could potentially provide a novel perspective on mechanisms of MBCT.

## MBCT From the Perspective of Bayesian Brain Theories

This section turns to the central question of this article: Could Bayesian brain theories help obtain a novel view on how MBCT works? This question is particularly relevant given that Bayesian theories of brain function are expressed in terms of formal cognitive process models, such as predictive coding and active inference, which have increasingly been used in recent years to describe and understand psychiatric disorders (33, 34, 96, 97). An analysis of the concepts underlying MBCT in terms of these models may therefore open up potential new ways of understanding why MBCT is effective in preventing relapse of depression. Furthermore, because Bayesian models like predictive coding make concrete suggestions of how these cognitive mechanisms are implemented physiologically, it may be possible to derive concrete and experimentally testable predictions about the neurophysiological processes that mediate MBCT effects.

MBCT considers cognitive reactivity and an overly strong engagement in the doing mode (or more specifically, the employment of the driven-doing mode) as central risk factors for the relapse of depression. MBCT specifically targets these risk factors by encouraging the practitioner

- to cultivate a different mode of mind (the being mode),
- to adopt a decentered perspective, and
- to reduce cognitive reactivity.

In the following, we examine how these three central processes in MBCT might be understood from a “Bayesian brain” view. This discussion is guided by the structure of a brain circuit that has been proposed as a possible architecture for predictive coding and active inference and includes a low-level reflex arc for action that is influenced by a higher hierarchical system for perception and metacognition (**Figure 4**). The circuit shown in **Figure 4** concerns the specific case of bodily perception (interoception) and regulation (homeostatic and allostatic control); however, the general principle equally applies to externally directed perception (exteroception) and control, only the anatomical circuits change (25). In the following, we retain the general structure of this hypothetical circuit but focus on specific parts and omit anatomical designations for clarity.

### The Being Mode

The being mode, which is cultivated throughout the MBCT program, is characterized by an ability to allow and accept whatever sensations arise, without giving in to the urge to judge or change the sensations (1, p.72). From a Bayesian brain perspective, this attitude can be understood as a particular style of perceptual inference that is caused by a change in the relative precision-weighting of prior beliefs and sensations. Specifically, the being mode would correspond to a perceptual inference style where the top-down influence of prior beliefs is reduced and the percept is dominated by the bottom-up influences of “raw” sensations (i.e., the likelihood). How could this perceptual inference style arise? To answer this question, it is helpful to return to the basic principle of belief updating in Bayesian models, as shown in **Figure 2**. Translating the equation into simple words and simplifying slightly, this principle can be understood as follows:

(2) change of expectation∝(precision of input/precision of prior belief)*prediction error

This says that, at any level of the hierarchy, the belief update (which leads to the posterior and thus the percept) is proportional to the prediction error, but weighted by a precision ratio. This ratio roughly corresponds to sensory precision (precision of the likelihood) divided by the precision of the prior belief. This makes intuitive sense: the more importance one assigns to the sensory input, the more weight a prediction error should carry, leading to larger belief updates. On the contrary, the more precise (narrow) a prior belief, the less inclined one would be to change it when receiving new information.

**Figure 6:**
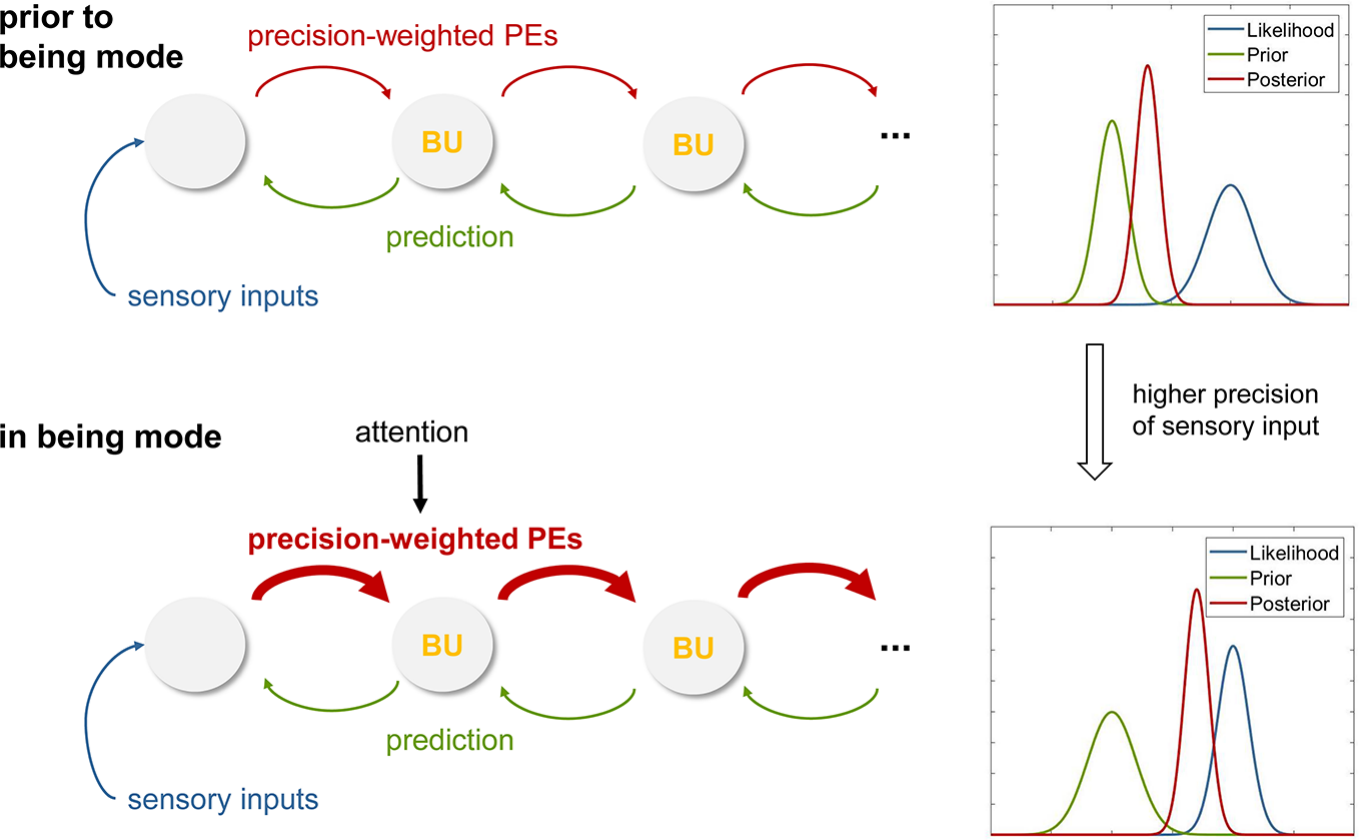
**(A)** Graphical summary of predictive coding that illustrates the exchange of prediction errors and predictions (prior beliefs) across levels of a cortical hierarchy. In this schematic of predictive coding, perception corresponds to Bayesian belief updates (BU) across the hierarchy. This panel represents the case before an individual adopts the being mode, with perception strongly shaped by priors (as illustrated on the right). **(B)** This panel illustrates a hypothetical mechanism for instantiating the being mode. Specifically, attentional modulation of forward connections in the cortical hierarchy is proposed to induce higher sensory precision and thus enhanced precision-weighting of prediction errors (PE), leading to rapid belief updates that are closely coupled to the sensory inputs. This corresponds to a perceptual style that lacks “bias” (as usually imposed by the brain’s internal model; compare panel A) and is closely synchronized to sensations. See main text for details.

Equation 2 predicts that a perceptual inference style where sensory information dominates and top-down influences by prior beliefs are weak can be achieved by increasing the precision of the sensory information (**Figure 6**). As a consequence, the beliefs at low levels of the processing hierarchy are updated rapidly and in close synchronization with the sensations, as are the percepts that result from these dynamically changing beliefs.

At this point, the question arises how an individual would be able to actively increase sensory precision. In theories of predictive coding, this is understood as an effect of attention: the precision of those sensory channels that are being attended to increases, while the precision of unattended channels decreases (98). Indeed, this matches the training phase of MBCT in which the practitioner learns to maintain and gently re-orient attentional focus to the sensations as they arise.

Neurophysiologically, attention-induced changes in precision are thought to rely on changes in neuromodulatory (particularly cholinergic) projections from the basal forebrain and the brainstem that alter the excitability of cortical neurons (32). Changes in excitability, in turn, determine the variability of activity in neuronal populations and alter the slope of the typical sigmoidal relationship between membrane potential and firing rate at the neuronal population level (99, 100).

In summary, this predictive coding view suggests that cultivating the being mode in MBCT could be understood as enhancing sensory precision at lower levels of cortical hierarchies and that this change in precision weighting could be achieved by the attentional focus that is acquired during MBCT practice.

### Decentering

**Figure 7:**
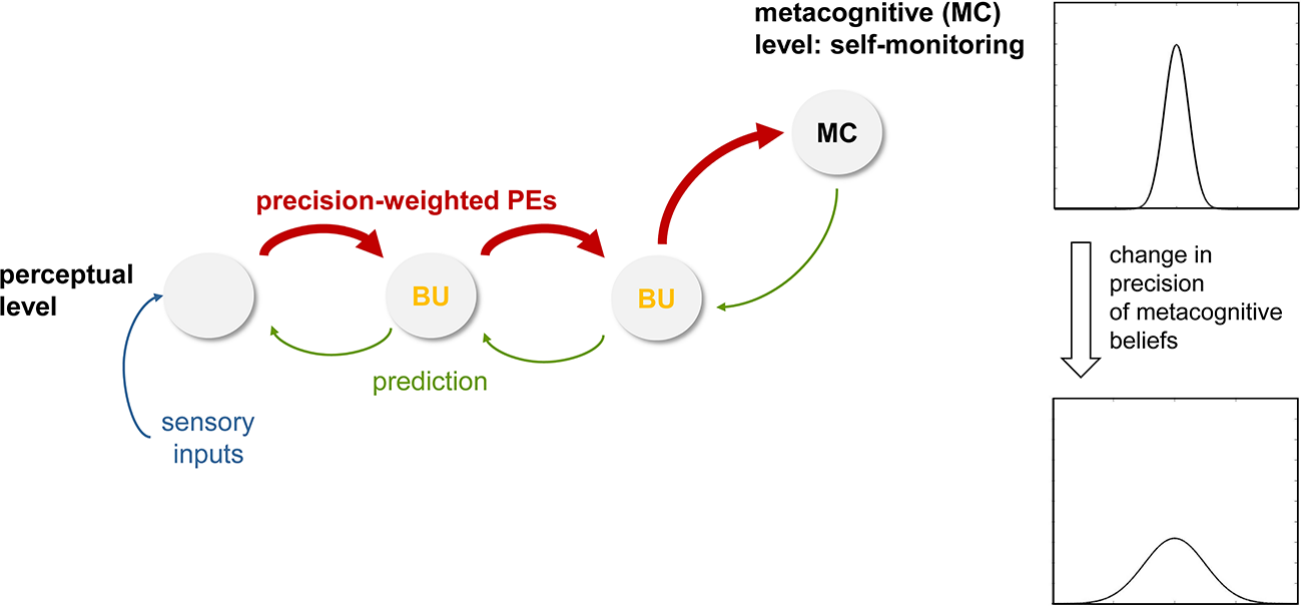
This figure illustrates a hypothetical mechanism for inducing decentering, within a hierarchical cortical network for perceptual inference plus an additional metacognitive layer at the top. Here, the notion is that during the being mode (with attentional modulation of forward connections, higher precision-weighting of prediction errors, PE, and rapidly ongoing belief updates, BU; compare **Figure 6**), high-level beliefs about one’s agency and level of control at the meta-cognitive level are altered and become less precise. See main text for details.

Following directly from above, the next step of analysis concerns decentering, the ability to experience thoughts and percepts simply as events in the mind that arise and pass. In the being mode, under the influence of high sensory precision induced by attention, prediction errors receive a large weight (compare Equation 2) and are carried up to higher levels where more abstract beliefs are represented. If the incoming sensations are variable, perceptual inference at higher levels undergoes dynamic changes from moment to moment as the incoming sensory information changes. If present for a prolonged period, these constant belief updates at higher levels of perception, triggered by the propagation of precise prediction errors up the hierarchy, may lead to the recognition—presumably at very high, metacognitive levels—that beliefs which were thought to be fixed and define the “self”— such as one’s ability to exert control and maintain prediction errors at a certain level (85)— can actually change (compare Session 6 of MBCT on “Thoughts are not Facts” in (1), p. 299). In other words, the individual’s attachment to high-order beliefs about one’s own agency and control, which were previously assigned high importance and were understood as integral to one’s identity, may loosen. Computationally, this could be represented by a reduced precision of beliefs about one’s agency and capacity of control (**Figure 7**). It is worth repeating that this process of decentering would not occur within the perceptual inference hierarchy itself, but at a higher (metacognitive) level where prediction errors within the perceptual hierarchy are monitored (**Figure 7**). This view continues to understand decentering as a metacognitive phenomenon—as is common in mindfulness concepts that refer to decentering as “meta-awareness” (101, 102)—but suggests a specific and novel form that views decentering as a direct metacognitive consequence of perceptual changes that occur during the being mode. It predicts that metacognitive predictions about the level of prediction errors encountered in the perceptual hierarchy should change (i.e., have less precision), and that this change should be reflected by altered connections from metacognitive to perceptual areas (see **Figure 7**). These areas are discussed in concrete anatomical terms below.

### Reactivity

**Figure 8:**
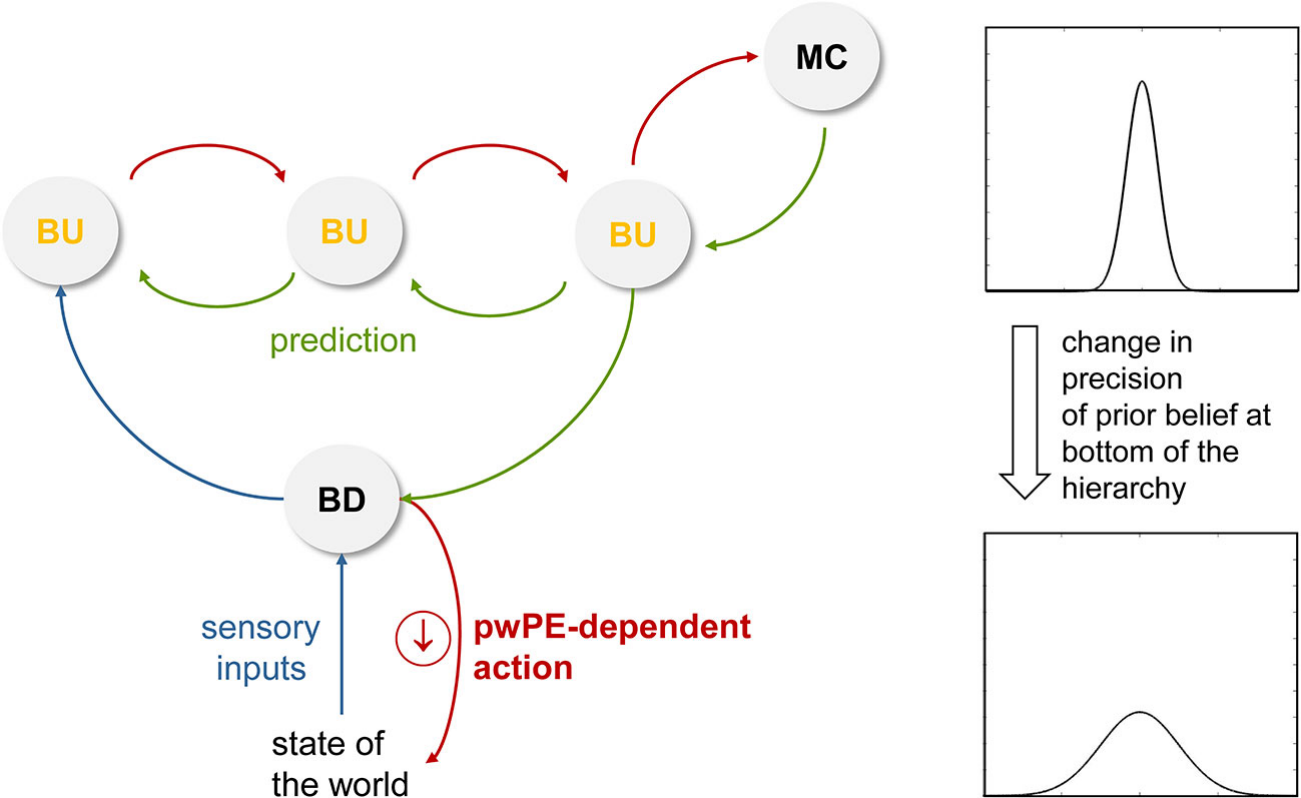
This figure illustrates a hypothetical mechanism for the reduction of reactivity. Here, a reduced precision of beliefs about the state of the world (e.g., bodily states) decreases the tendency to react to any discrepancy between the sensory inputs expected under this belief and the actual sensory inputs (prediction error). This is because the vigor of reflex-like actions that are emitted to “defend” beliefs depends on precision-weighted prediction error (see Stephan et al. (85) for mathematical details). pwPE, precision-weighted prediction errors; BU, belief updates; BD, belief defending. Compare **Figure 4** for a (hypothetical) anatomical circuit and see main text for details.

Finally, Bayesian brain concepts can also help to understand cognitive and physiological reactivity in a novel way. Specifically, from an active inference view, (re)actions serve to fulfil prior beliefs in order to reduce prediction error. This is perhaps most easily discussed by using bodily regulation as an example (but can equally be extended to cognitive processes). If sensory inputs from the body deviate from prior beliefs about expected bodily states (homeostatic beliefs), actions are deployed in a reflex-like fashion. Importantly, as described by Bayesian treatments of homeostatic control (85), the higher the precision of the homeostatic belief, the greater the significance of a prediction error and the more forcefully an action is executed in order to reduce it. This reflex-like mechanism, however, is thought to be under control by higher-order beliefs (e.g., predictions from the perceptual hierarchy or a change in metacognitive beliefs; see **Figures 4** and **8**) that can alter the properties of homeostatic beliefs and thus induce anticipatory action (85). This may involve a shift in the expectation (mean) of prior beliefs or a change in their precision [compare **Figure 6** in (85)]. Similar to the proposal in Seth and Friston (103), this might be implemented physiologically by descending projections (e.g., from anterior cingulate; **Figure 4**) that alter local excitation-inhibition balance and change the variability of activity in the neuronal population that encodes the homeostatic belief (e.g., in the hypothalamus or brainstem). Flattening the prior in this manner would reduce homeostatic reflexes and decrease the reactive tendency to respond to changes in incoming sensations.

Moving beyond homeostatic regulation, the same principle of action vigor depending on the precision of prior beliefs that the actions are meant to fulfil has been proposed to hold in general (25). If this assumption turns out to be correct, reduction of cognitive reactivity may thus be seen as a decrease in the precision of prior beliefs: for such “flat” priors, the range of expected states of the world becomes broad, which renders any sensation that deviates from the prior’s mean (expectation) less meaningful and decreases the impulse to respond to incoming sensations (see **Figure 8**).

Applying this general notion to MBCT, under the perspective presented in this paper, it is conceivable that a decrease of prior precision could be a consequence of the cultivation of the being mode and the adoption of a decentered perspective. More specifically, following a period of heightened prediction error signals and constant belief updates (**Figure 6**) and the metacognitive insight that even the most high-level beliefs may undergo dynamic changes (**Figure 7**), a reduction of reactivity due to decreased precision of the prior may constitute a consequence of cultivating the being mode and having adopted a decentered perspective. In other words, experiencing constant belief updating in the perceptual hierarchy and adjusting one’s metacognitive expectations such that higher magnitudes of prediction errors become “acceptable” (i.e. expected) may subsequently invoke a lessened tendency to defend beliefs against perturbations. This could be implemented by top-down influences from perceptual and metacognitive areas onto low-level effector regions and would extend the cognitive changes induced by MBCT to the domain of responding, reducing the emission of reflex-like reactions to stimuli, thoughts, or emotions (**Figure 8**).

## Testing the Theory’s Implications for MBCT Experimentally

**Figure 9:**
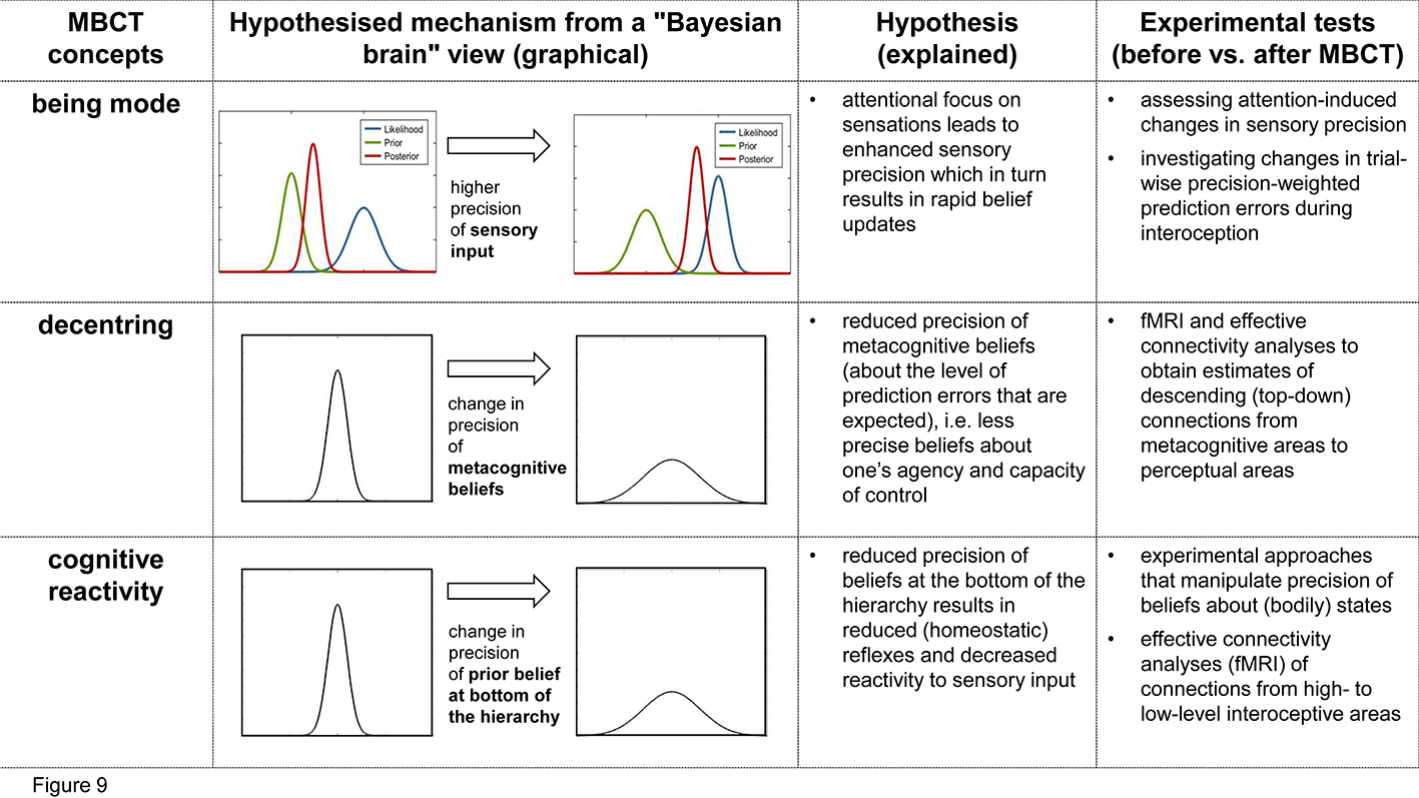
Summary of the hypotheses presented in this paper and the proposed experimental tests. This figure relates key concepts from MBCT (first column) to a proposed Bayesian brain perspective of its mechanisms (second column), a brief summary of this hypothesis (third column), and possible experimental tests (fourth column).

The previous section described three proposed mechanisms how core components and processes in MBCT—being mode, decentering, and reduced reactivity—could be understood from the perspective of Bayesian models of brain function. Clearly, so far, the above accounts of MBCT are speculative and purely theoretical. Importantly, however, since the Bayesian brain framework that our view on MBCT is grounded in makes concrete suggestions of how cognitive processes are implemented physiologically, we can derive experimentally testable predictions. In the following, the predictions that originate from the above discussion and suitable experimental tests are described briefly (see **Figure 9** for a summary of our hypotheses and proposed experimental tests). The suggestions below refer to longitudinal designs that compare participants before and after exposure to MBCT, i.e., the standard 8-week program. In other words, we focus on predicted *changes* in computational and neurophysiological processes that occur over the duration of the MBCT program and that could be assessed in pre-MBCT vs. post-MBCT comparisons.

Our first proposal above was that the being mode is characterized by a shift of attentional focus that leads to enhanced sensory precision (**Figure 6**). This hypothesis—and its implication that enhanced sensory precision weighting should develop over the period of MBCT training—can be tested using established experimental procedures for assessing attentionally induced changes in precision. For example, Petzschner et al. (104) found that a late component of the heart-beat evoked potential (HEP) – an event-related EEG signal that is thought to reflect precision-weighted prediction error in interoception—is altered by attentional focus towards vs. away from the heart. Importantly, the paradigm by Petzschner et al. (104) provides a quantitative measure of attentional modulation of the HEP amplitude (the ΔHEPa index) and thus changes in sensory precision. This would render it a useful tool to investigate the predicted increase in sensory precision after completion of an MBCT program, compared to before. Our hypothesis would be rejected if, in patients who successfully completed the MBCT program and clinical improvement, we failed to find the predicted increase in ΔHEPa. This failure could be detected according to conventional criteria of Bayesian statistics, i.e., a Bayes factor of three or larger in favor of the “null hypothesis” of no change.

Another possibility would be to use computational modelling to obtain subject-specific estimates of precision-weighted prediction errors during interoception in a trial-by-trial manner. Specifically, one could test whether, after completion of the MBCT program, the precision of prediction errors about bodily states is increased when subjects switch into the being mode. Estimates of trial-wise precision weights can be obtained, for example, using hierarchical Bayesian models, such as the Hierarchical Gaussian Filter [HGF; (105, 106)] which has previously been used to infer dynamic changes of precision from behavioral or physiological data [see (36, 107, 108)]. One technical challenge of this approach in the interoceptive domain is that one requires repeated and controlled perturbations of bodily states in a safe and non-invasive manner (see discussion below).

Our second proposal above was that decentering corresponds to a change in metacognition, effectively leading to less precise beliefs about the level of prediction errors that are expected to be encountered. In the model shown by **Figure 7**, this would manifest as a change in the descending (top-down) connections from metacognitive areas to perceptual areas that are thought to signal these metacognitive beliefs (**Figure 7**). In principle, this is testable by fMRI and effective connectivity analyses that allow for obtaining directed estimates of connections [e.g., (109)]. However, one caveat is that it is not perfectly understood how a less precise belief translates into the strengths of descending connections. It seems plausible to assume that descending connection strengths should be reduced, however, to our knowledge, this question has not been examined so far. For the moment, our hypothesis therefore only refers to changes per se in descending connections from metacognitive areas to perceptual areas, not to the sign of these changes.

Previously, metacognitive processes have only been examined in terms of functional connectivity which does not allow for interpreting the directionality of connections (110, 111). Testing our proposal would require effective connectivity analyses that compare the connectivity of metacognitive areas before and after the completion of an MBCT program. Concretely, in the exteroceptive domain, this would correspond to examining connectivity from frontopolar Brodmann area 10 in anterior prefrontal cortex (112) to areas at the top of sensory hierarchies. In the interoceptive domain, an area that is likely placed at the top of the interoceptive hierarchy (and also receives exteroceptive information) is the anterior insula [see Seth (113) and compare **Figure 4**]. By contrast, the anatomical area(s) implementing metacognition are not well known so far; however, a plausible candidate region is the medial anterior prefrontal cortex [see the discussion in Stephan et al. (85)].

Our third proposal concerned the reduction of reactivity due to MBCT and consisted of two components. First, we postulated that a decrease in reactivity would result from reduced precision of homeostatic beliefs thought to be encoded in lower visceromotor regions that trigger regulatory actions in a reflex-like fashion (e.g. hypothalamus or brainstem nuclei like the periaqueductal grey, PAG; see **Figure 4**). Second, we suggested that a reduction in homeostatic belief precision could be caused by descending connections from high-level interoceptive areas (such as anterior insula, AI, or anterior singlet cortex, ACC) that might transmit allostatic predictions (compare **Figure 4**). Testing the first component of this proposal could be achieved by experimental approaches that reduce the precision of beliefs about bodily states in a controlled and predictable manner and then test whether these changes are reflected by changes in bodily states and by activity in visceromotor brain regions like the hypothalamus or PAG (114). This approach has recently been pioneered by Grahl, Onat (115) in the context of placebo studies.

Testing the second component of this proposal would require effective connectivity analyses and fMRI. Specifically, one would need to assess whether an MBCT-induced reduction of reactivity to experimentally controlled perturbations of homeostasis would be related to a change in connectivity from cortical areas assumed to compute allostatic predictions (like AI and ACC) to low-level visceromotor regions. Here, the same caveat applies as for tests of the second proposal, i.e., the theory presently only allows for predicting changes in connectivity per se, but not the sign of these changes. Furthermore, a significant technical challenge is to choose suitable techniques for perturbing homeostasis safely and repeatedly during an experimental session. This could involve approaches like auricular (percutaneous or transcutaneous vagus nerve stimulation; 116), short-lived pharmacological (117) or immunological (118) interventions, or manipulations of cardiac and respiratory processes (119). For a more detailed discussion of this challenge and potential solutions, see Khalsa et al. (120) and Critchley and Garfinkel (121).

## Conclusions

In this paper, we have described how core components of MBCT—i.e. the being mode, decentering, and cognitive reactivity—can be understood in terms of mechanisms that derive from Bayesian brain concepts. The mechanisms we propose essentially concern changes in precision-weighting (of sensory inputs and various beliefs, respectively) that are elicited by the elements of the MBCT program and concern different levels of perceptual hierarchies (see **Figure 9** for a summary).

We have outlined how our hypotheses regarding mechanisms of MBCT could be tested empirically. Clearly, this proposal represents an initial blueprint and will undoubtedly experience revisions in the future as the anatomical nature of the hierarchies involved and the available models to represent hierarchical Bayesian become more concrete and refined. Nevertheless, we hope that this article already illustrates that a Bayesian perspective on MBCT not only offers exciting opportunities to better understand the processes that MBCT elicits, but that the proposed explanations can also be tested experimentally. If the ideas presented in this paper turn out to have substance, they may be useful for informing further developments of MBCT and eventually for understanding the variability across patients with regard to individual treatment response.

## Author Contributions

Z-MM wrote the initial version of this manuscript. SI contributed and edited.

## Funding

Z-MM acknowledges support by the Wilhelm Schulthess Foundation.

## Conflict of Interest

The authors declare that the research was conducted in the absence of any commercial or financial relationships that could be construed as a potential conflict of interest.
